# Neutrophil dysregulation differentiates pediatric septic shock biomarker-based mortality-risk strata: insights from weighted gene co-expression network and transcriptomic analyses

**DOI:** 10.3389/fimmu.2025.1663704

**Published:** 2025-11-19

**Authors:** Leland Dunwoodie, Min Huang, Andrew R. Moore, Natalja L. Stanski, Stephen W. Standage, Jennifer M. Kaplan, Basilia Zingarelli, Kelli Harmon, Julie C. Fitzgerald, Scott L. Weiss, Michael T. Bigham, Adam J. Schwarz, Riad Lutfi, Neal J. Thomas, Bereketeab Haileselassie, Parag N. Jain, Timothy E. Sweeney, Rishikesan Kamaleswaran, Mihir R. Atreya, Andrew J. Lautz

**Affiliations:** 1 Division of Critical Care Medicine, Cincinnati Children’s Hospital Medical Center, Cincinnati, OH, United States; 2 Department of Biomedical Informatics, Emory University School of Medicine, Atlanta, GA, United States; 3 Stanford Institute for Immunity, Transplantation and Infection, Stanford University School of Medicine, Stanford, CA, United States; 4 Center for Biomedical Informatics Research, Department of Medicine, Stanford University School of Medicine, CA, United States; 5 Department of Pediatrics, University of Cincinnati College of Medicine, Cincinnati, OH, United States; 6 Children’s Hospital of Philadelphia, Philadelphia, PA, United States; 7 Nemours Children’s Hospital, Wilmington, DE, United States; 8 Akron Children’s Hospital, Akron, OH, United States; 9 Children’s Hospital of Orange County, Orange, CA, United States; 10 Riley Hospital for Children, Indianapolis, IN, United States; 11 Penn State Hershey Children’s Hospital, PA, United States; 12 Lucile Packard Children’s Hospital Stanford, Palo Alto, CA, United States; 13 Heart Center, Children's Health, Division of Cardiology, Department of Pediatrics, University of Texas Southwestern Medical Center, Dallas, TX, United States; 14 Inflammatix, Sunnyvale, CA, United States; 15 Department of Surgery, Duke University School of Medicine, Durham, NC, United States

**Keywords:** precision medicine, pediatric sepsis, mortality risk stratification, transcriptomics, immune response, neutrophil dysregulation

## Abstract

**Background:**

Pediatric sepsis is a leading cause of global mortality, particularly among children, with limited therapeutic options beyond antibiotics and organ support. The Pediatric Sepsis Biomarker Risk Model (PERSEVERE-II) stratifies mortality risk in pediatric septic shock, yet the molecular mechanisms underlying high mortality risk remain incompletely understood.

**Methods:**

We analyzed whole blood transcriptomes collected from 81 children with septic shock on day 1 of meeting study criteria. Patients were stratified into high- and low-mortality risk groups according to the PERSEVERE-II biomarker risk model. Using weighted gene co-expression network analysis (WGCNA) and differential gene expression analyses, we identified molecular pathways and transcription factors (TFs) associated with mortality risk. Cell type differences were inferred using CIBERSORTx and using a reference single-cell dataset inclusive of neutrophils and their subsets.

**Findings:**

We identified distinct molecular profiles with high-risk patients displaying significant overexpression of genes related to neutrophil degranulation and innate immunity, alongside suppressed adaptive immune responses. The predominance of developing neutrophils underscored a major role of emergency granulopoiesis. Key TFs identified, including *LTF*, *FOXM1*, *KLF1*, and *CEBPB*, were linked to high-risk gene expression signatures. Our findings indicate a pathological shift toward a dysregulated neutrophil-driven hyperinflammation and adaptive immune suppressive state, which together are associated with adverse outcomes.

**Interpretation:**

Our results suggest that neutrophil dysregulation underpins the high mortality risk conferred by the PERSEVERE-II model. The identified transcriptional regulators may provide potential targets to mitigate neutrophil dysregulation and improve outcomes among high-risk patients.

## Introduction

Sepsis is a heterogenous disease associated with high morbidity and mortality worldwide (1). Notably, 40% of sepsis cases occur in children under five years (2), making sepsis the leading cause of mortality in this age group, responsible for 20% of all under-five deaths (3). In the United States alone, pediatric sepsis claims over 7,000 lives annually (4) and incurs $7 billion in hospitalizations (5). Despite this burden of disease, treatments remain limited to antibiotics and organ function support as clinical and biological heterogeneity among critically ill children with sepsis continues to hamper the identification of efficacious therapies (6). Over the past two decades, using a precision medicine framework research has aimed to address this heterogeneity, striving to match the right therapy with the right patient at the right time (7).

The Pediatric Sepsis Biomarker Risk Model (PERSEVERE) was developed to stratify children with septic shock based on mortality risk (8). PERSEVERE-II built upon the original model by incorporating admission platelet count along with five protein biomarkers measured in sera collected within 24 hours of the onset of septic shock in children admitted to pediatric intensive care unit (PICU) to assign a 28-day mortality probability (9). Both models have been prospectively validated in observational cohorts of children with septic shock (10). While PERSEVERE-II reliably estimates mortality risk, it remains unclear what molecular features underlie children at high risk as compared to those at low risk of mortality. It follows that a comprehensive assessment of the underlying pathobiology could inform the development of targeted interventions specific to high-risk patients.

We utilized whole blood transcriptomic data from pediatric septic shock patient to conduct weighted gene co-expression network analyses (WGCNA) to identify genes associated with high PERSEVERE-II mortality risk, with functional annotations highlighting neutrophil-related processes. We identified differentially expressed genes distinguishing high- versus low-mortality risk patients, as stratified by the PERSEVERE-II biomarker model. Biological pathway analyses indicated overexpression of innate immune responses with concurrent repression of adaptive immune responses early in the illness course distinguished high-risk patients. We employed computational tools to identify transcription factors regulating implicated genes and applied deconvolution algorithms alongside reference single-cell data to identify cell subpopulations contributing to mortality risk. Taken together, our findings align with prior studies suggesting developing neutrophils and emergency granulopoiesis (11) contribute to a hyperinflammatory state linked to adverse septic shock outcomes.

## Methods

### Patient enrollment

This study leveraged biospecimens from the Sepsis Genomics Collaborative –a prospective multi-center observational cohort, which has been previously detailed extensively (12–15). Briefly, critically ill children between the ages of 1 week and 18 years who met consensus criteria for septic shock (16) were included and enrolled from 13 pediatric intensive care units (PICUs) across the United States from May 2015, through February 2019. The study protocol received approval from the Institutional Review Boards (IRBs) of the primary site (Cincinnati Children’s Hospital IRBs, Genomics of Septic Shock, IRB ID: 2008–0558 and 2022-0721) and all participating institutions. Informed consent was obtained from parents or legal guardians. All procedures involving human participants adhered to the ethical standards of the participating institutions’ IRBs, the 1964 Helsinki Declaration and its subsequent amendments. Whole blood collected in PAXgene RNA tubes and sera collected within 24 hours of the onset of septic shock were used. No study-related interventions occurred beyond these blood draws. De-identified clinical data were collected daily from days 1 to 7 of PICU admission, with mortality data tracked up to 28 days post-enrollment.

### PERSEVERE biomarker measurement and risk-stratification

In addition to PICU admission platelet count, the 5 PERSEVERE biomarkers interleukin 8 (IL-8), heat shock protein (HSPA1B), granzyme B (GZMB), matrix metalloprotein 8 (MMP8), and C-C motif chemokine ligand 3 (CCL3) were previously measured in day 1 sera, permitting patient assignment to one of nine PERSEVERE-II Terminal Nodes (TNs) based on the published classification and regression tree (CART) (9, 10). Patients classified to TN 1, 2, 5, and 8 were predicted to be survivors and designated as low risk (<1.9% risk of death). Patients classified to TN 3, 4, 6, 7, and 9 were predicted non-survivors and considered high risk for mortality (16.7%-44.4% risk of death) (10). Demographic and clinical characteristics were compared between children stratified to low and high PERSEVERE-II risk. Pediatric Risk of Mortality (PRISM)-III scores were evaluated as an estimate of baseline illness severity (17). Immunocompromised status, vasoactive and corticosteroid use, prevalence of mechanical ventilation and renal replacement therapy were recorded. Outcome variables included PICU length of stay, PICU-free days, hospital length of stay, 7- and 28-day mortality, and the prevalence of complicated course. Complicated course was defined as the persistence of at least two organ failures at 7 days or mortality by 28 days. Dichotomous variables were compared with the Fisher exact test or chi-squared test. Nonparametric continuous variables were characterized as medians with interquartile ranges (IQRs) and evaluated with the Wilcoxon rank-sum test.

### RNA extraction and library preparation

Whole blood was collected in PAXgene Blood RNA tubes and stored at −80 °C. For processing, tubes were thawed at room temperature for 2 h, inverted to homogenize, and 3 mL aliquots were transferred. RNA was isolated using a modified RNeasy Mini protocol on a QIAcube (QIAGEN). Briefly, PAXgene blood/stabilizer was diluted with PBS and centrifuged at 3,000 × g to pellet nucleic acids; pellets were washed with nuclease-free water, re-pelleted (3,000 × g), resuspended in Buffer RLT, treated with Proteinase K, and passed through gDNA-elimination columns. Flow-through was combined with isopropanol, bound to a MinElute column, washed with 80% ethanol, and eluted in RNase-free water. Eluates were heat-denatured (55°C, 5 min) and snap-cooled. RNA quantity was measured by Qubit RNA assays and integrity by BioAnalyzer; samples with RIN < 7 were excluded. Globin RNA was removed with GLOBINclear (Invitrogen) per manufacturer’s instructions. Globin-depleted RNA was quantified (Qubit RNA HS), and 10 ng was used for rRNA depletion and library construction with the SMARTer Stranded Total RNA-seq Kit v2—Pico Input Mammalian (Takara). Libraries were quantified (Qubit dsDNA HS), sized (Fragment Analyzer High Sensitivity Small Fragment kit), pooled, and sequenced on an Illumina NovaSeq 6000 (paired-end, 2 × 100 bp). Per sample, 40–120 million read pairs were generated. FASTQ files were used for downstream processing. Library prep and sequencing were performed at TB-SEQ (Palo Alto, CA).

### Gene expression matrix

Raw mRNA counts were mapped to 60,846 Ensembl Gene IDs across samples. The Gene IDs which did not correspond to a known Human Genome Gene Symbol and Entrez ID were removed, leaving 20,239 genes. In addition, 171 Ensembl Gene IDs were true duplicates with identical mRNA count data and were removed. There were nine duplicate Human Genome Gene Symbol pairs, each of which had one member that was less expressed than the other and removed. Finally, there were 26 unique Entrez IDs corresponding to two or three Gene Symbols, 55 Gene Symbols in all. These were manually evaluated, and the Gene Symbol with the lowest average expression was removed. Altogether, this left a gene expression matrix with 20,030 genes across 81 samples.

### Weighted gene co-expression network construction

We conducted Weighted Correlation Network Analysis (WGCNA) (18) to identify gene-modules, representing co-expressed genes, associated with biomarker mortality risk strata and to explore relationships among genes. First, the raw gene expression matrix was normalized using trimmed-mean of M-values (TMM) (19) and low-expression values were removed using edgeR (20) using the default min.count = 10, leaving 13,515 genes. This normalized, log2-transformed matrix was used for network construction. A WGCNA soft threshold of 12 was selected with R^2^ = 0.817 and mean connectivity = 70.200, meeting our goals of scale free topology model fit coefficient > 0.80 and mean connectivity < 100 to achieve maximum correlation strength in addition to appropriate hub connectivity for analysis. (**Supplementary eFigure S1**). Pearson’s correlation coefficients were calculated to assess the strength of correlation. Signed network construction was utilized to emphasize directional correlation relationships between genes. For each gene co-expression module of interest, its gene expression between conditions was evaluated by comparing module eigengene expression for high- and low- mortality risk patients. WGCNA calculates an eigengene expression value for every patient across every module which represents the first principal component of the gene expression for that patient across all genes in that module. The eigengene expression of high-risk and low-risk patients were compared using a two-tailed heteroscedastic t-test. WGCNA also computes each gene’s correlation with its module and the significance of that correlation; the genes in each module whose correlation with that module has the most significant p-value were identified as driver genes. Similarly, WGCNA calculates each gene’s correlation with the trait of interest and the significance of that correlation, which revealed the genes whose expression was most associated with the high-risk strata. The gene lists in modules of interest were submitted to the Database for Annotation, Visualization and Integrated Discovery (DAVID) to identify functional annotations (21); with only those with a Benjamini-Hochberg adjusted p-value < 0.05 being considered significant.

### Differential gene expression, functional pathway annotation, and upstream regulators

We identified differentially expressed genes (DEGs) comparing high- and low-mortality risk patients using R package DESeq2 (22). We used a Benjamini-Hochberg adjusted false discovery rate (FDR) threshold of 0.05 to identify DEGs. Heatmap and volcano plots were used to visualize DEGs. The biological relevance of pathways were determined based on Gene Ontology (GO) annotations and Kyoto Encyclopedia of Genes and Genomes (KEGG) using clusterProfiler (22).

### Inferring differences in cell types associated with risk-strata

We inferred relative differences in cell type abundance comparing risk-strata using CIBERSORTx (23) based on differentially expressed genes. However, this computational tool was originally designed for *in silico* tissue deconvolution rather than blood and lacks a reference for cell types specific to critically ill patients. To address this limitation, we used single-cell RNA sequencing dataset comprised of critically ill adults with sepsis published by *Kwok* et al. (11) We calculated a composite gene score as the geometric mean of top 20 overexpressed genes minus the geometric mean of top 20 repressed genes using published methods (24), identified through DEG analyses comparing risk-strata and also available in the single-cell dataset. We mapped this scaled composite score against the Uniform Manifold Approximation and Projection (UMAP) of the *Kwok* dataset to infer cell types contributing to biological differences between risk-strata.

### Intercellular communication analyses among risk-strata

We used the *Kwok* dataset to generate pseudobulk gene-expression data. For each individual patient represented in this dataset, gene-expression in each cell type was aggregated using the AggregateExpression function in Seurat. The pseudobulk matrix was batch-corrected using the ComBatseq function from the sva package (v.3.46.0), a negative binomial regression method. DEGs comparing risk strata, identified previously, were selected as features for downstream analyses. To assign risk strata in the reference single cell dataset, we developed a Support Vector Machine (SVM) classification model. The normalized matrix and corresponding labels were randomly split into training and validation sets (80:20 ratio) to train and fine tune the SVM model. This model was applied to the corrected pseudobulk data to assign high- or low-risk labels to all single-cell sepsis samples. Finally, CellChat analysis (v2.1.0) was performed on the single-cell matrix, to infer cell-cell communication networks focusing on the high-risk strata.

### Identification of transcription factors

To identify key transcriptional regulators among high-risk patients, we submitted gene lists in each WGCNA module with statistically significant association with high-risk strata to the Chip Enrichment Analysis (ChEA3) portal (https://maayanlab.cloud/chea3/) to predict transcription factors (TFs) anticipated to regulate gene co-expression module (25). The most notable transcription factor (TF) is denoted by the lowest mean rank, which indicates the TF predicted by ChEA3 to interact most with the submitted gene lists after searching across multiple libraries including ENCODE, GTEx, ARCHS4, and ReMap. Additionally, we submitted DEGs distinguishing patient risk-strata for Ingenuity Pathway Analysis (IPA, QIAGEN) (26) (QIAGEN) to identify upstream regulators and mechanistic networks that could influence gene expression patterns, focusing on direct interactions between regulators and selecting TFs with highest activation z-scores and p<0.001.

## Results

A total of 81 patients were included in the study, of whom 24 patients were designated as high risk and 57 patients as low risk for mortality according to the PERSEVERE-II stratification tool. Demographic, clinical, and outcome variables comparing patients in each of the risk-strata are detailed in **Table 1**. Patients classified as high risk were more severely ill at illness onset, and had greater mortality at 7 and 28 days, as well as a greater burden of complicated course, relative to low mortality-risk patients.

**Table 1:** Demographic, clinical characteristics, and outcomes comparing patients classified as high vs. low mortality risk based on the pediatric sepsis biomarker risk model II (PERSEVERE-II). Data presented as n (%) or median (IQR) as appropriate.

Variable	High-mortality risk group (n=24)	Low-mortality risk group (n=57)	P value
Age (years)	2.8 (0.8, 6.1)	4.9 (1.5, 5.6)	0.104
Sex (female)	12 (50.0%)	32 (56.1%)	0.612
Race			0.529
White or Caucasian	19 (79.2%)	42 (73.7%)	
Black or African American	3 (12.5%)	5 (8.8%)	
Other	2 (8.3%)	10 (17.5%)	
Ethnicity			0.366
Hispanic or Latino	3 (12.5%)	12 (21.1%)	
Co-morbidity	11 (45.8%)	27 (47.4%)	0.899
Immunocompromised	2 (8.3%)	3 (5.3%)	0.600
PRISM-III	17 (11, 27)	8 (3, 12)	<0.001
Vasoactive use	24 (100%)	51 (89.5%)	0.099
Corticosteroid use	18 (75.0%)	32 (56.1%)	0.111
Mechanical Ventilation	24 (87.5%)	44 (77.2%)	0.287
Renal Replacement	3 (12.5%)	3 (5.3%)	0.257
PICU LOS	8 (3, 14)	7 (3, 11)	0.889
PICU Free Days	20 (14, 25)	21 (18, 25)	0.889
Hospital LOS	15 (10, 20)	14 (9, 25)	0.918
7-day mortality	4 (16.7%)	0 (0%)	0.002
28-day mortality	6 (25.0%)	0 (0%)	<0.001
Complicated course	11 (45.8%)	11 (19.3%)	0.014

### Weighted gene co-expression network analyses identifies four modules associated with high-mortality risk strata

**Figure 1:**
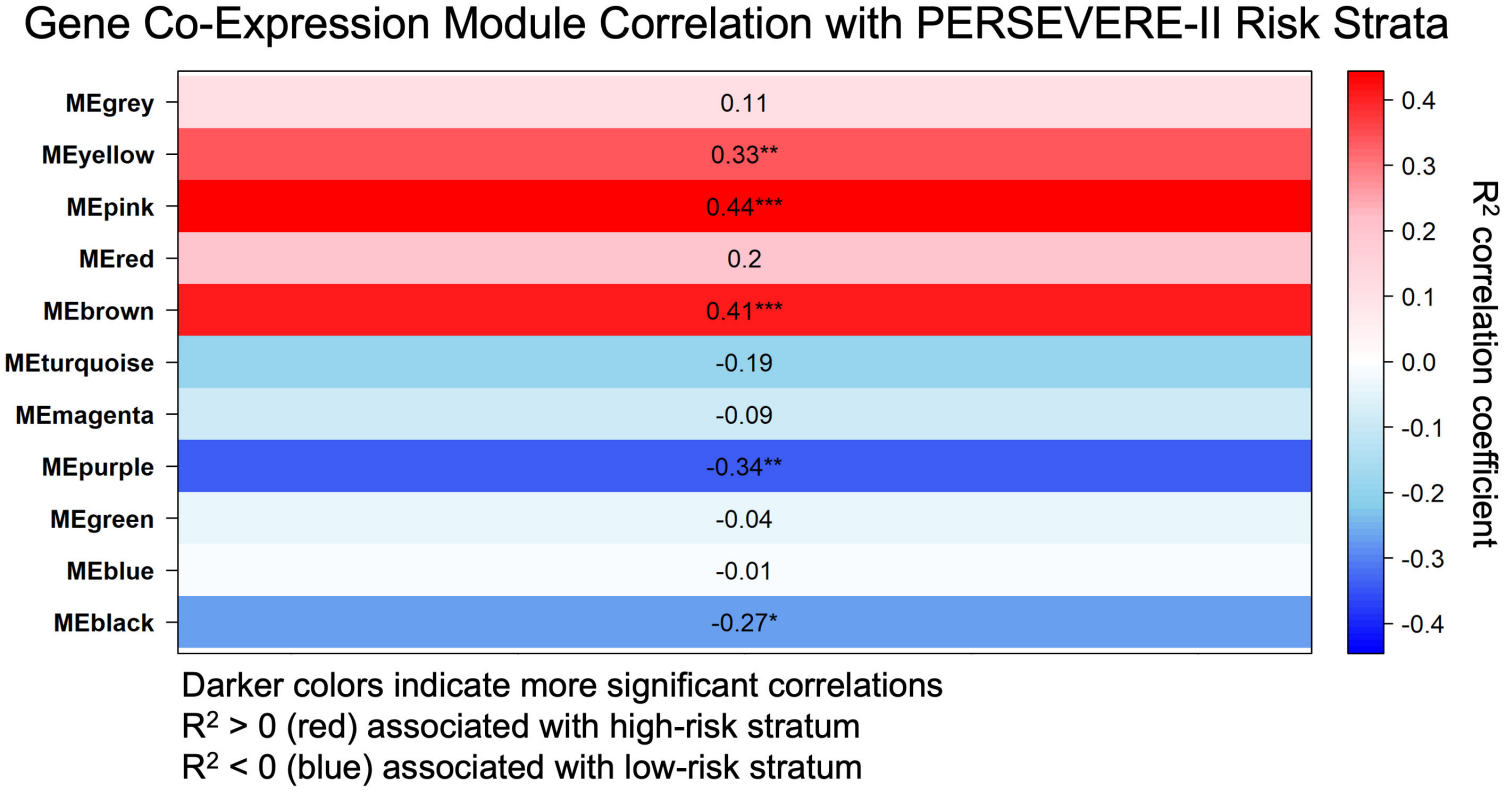
Weighted gene co-expression network analysis (WGNCA) of gene expression data from children at high and low risk of sepsis mortality based on pediatric sepsis biomarker risk model II (PERSEVERE-II) identified the Pink Module of genes (MEpink or Module Eigengene Pink) as the most correlated with the high-risk phenotype based upon correlation coefficient (R^2^ = 0.436) and statistical significance (p = 4.795e^-5^). R^2^ correlation coefficient with the high-risk trait is shown, as is the p-value for the significance of that correlation, indicated by ***(p < 0.001), **(p < 0.01), or *(p < 0.05).

We identified 11 gene co-expression modules using Weighted Correlation Network Analysis (WGCNA). The correlation between these modules and the high-risk strata was calculated, as was the significance level corresponding to each correlation as shown in **Figure 1**. Fifty-one genes (designated the Pink module; **Supplementary Table S1a**) had the highest correlation (R^2^ = 0.436) and the most significant p-value for the correlation with the high-risk mortality strata (p = 4.795e^-5^). Notably, the gene most strongly associated with this module was *MMP8* (p = 1.53e^-36^), which encodes for one of the PERSEVERE-II biomarkers. The 5th- and 6th-most strongly associated genes with this module, *LCN2* and *RETN*, respectively, were two of the original 12 candidate PERSEVERE biomarker genes that were pruned from the final model. The gene *OLFM4*, previously associated with neutrophil subpopulations in pediatric septic shock (27), also has a strong association (p = 7.91e^-15^). Three Reactome pathways were significantly associated with this module: “Neutrophil Degranulation,” “Innate Immune System,” and “Immune System.” Three other gene-modules associated with mortality-risk strata are detailed in **Supplementary Tables S1b-d**. Comparison of gene module eigengene expression between mortality-risk strata is shown in **Supplementary eFigure S2**, with this 51-gene (“Pink”) module once again showing the greatest difference between groups.

We identified 260 genes that were significantly associated with the high-risk strata at p < 0.001 independent of their gene co-expression module membership (**Supplementary Table S2**). There was a high degree of overlap between these genes and DEGs identified by DESeq2, detailed subsequently. For example, the top seven genes identified by WGCNA associated with the high-risk cohort were identified by DESeq2 as genes with significantly lower expression in high-risk patients (*NHSL2*: p < 1.9e-11, *LRMP*: p < 4.14e-10, *IL16*: p < 6.16e-9, *GLIPR1*: p < 7.65e-9, *SRPK2*: p< 7.73e-9, *AOAH*: p< 1.33e-9, *CALB1*: p< 9.55e-9, **Supplementary Table S4a**). In addition, examining co-expression modules associated with high-risk strata, there were 9 Pink Module genes, 6 Yellow Module genes, and 24 Brown Module genes identified by WGCNA as significantly associated with the high-risk strata independent of module membership. All of these 39 genes were also shown to be over-expressed among high-risk patients with a p < 0.001 by DESeq2 (**Supplementary Table S3**).

### Differential gene expression analyses corroborate WGCNA analyses implicating neutrophil dysregulation among high mortality risk patients

**Figure 2:**
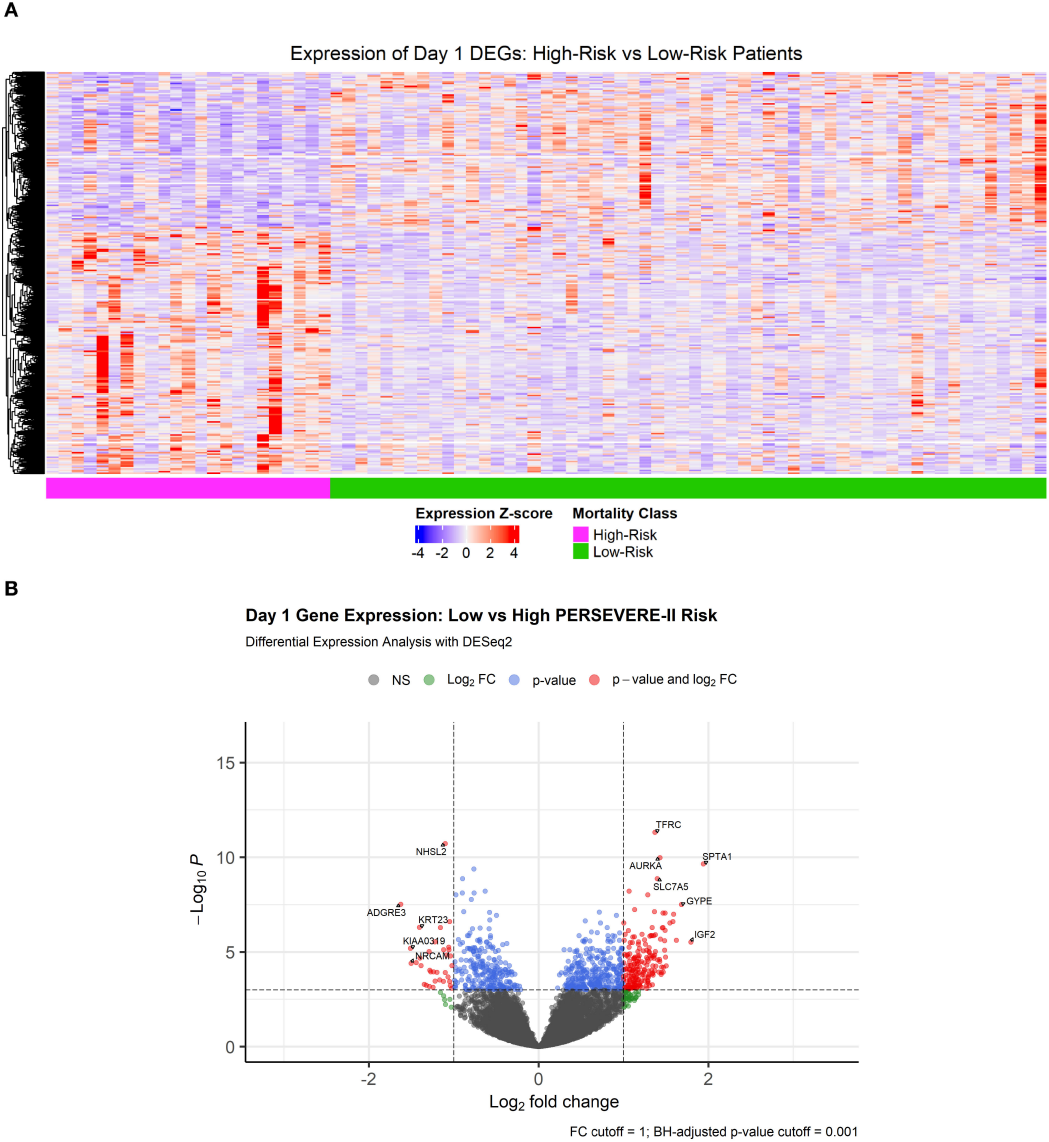
**(A)** Heatmap showing 2,654 differentially expressed genes at a Benjamini-Hochberg adjusted p-value < 0.05 comparing transcriptomic profiles of children with high- and low-mortality risk based on PERSEVERE-II model. **(B)** Volcano plot showing DEGs with a |log2FoldChange| > 1 and Benjamini-Hochberg adjusted p-value < 0.001.

Heatmap visualizing differentially expressed genes comparing high vs low mortality-risk patients is shown in **Figure 2a**. A total of 2,654 genes (13.3% of all sequenced genes) were differentially expressed at an adjusted p-value < 0.05, of which 1,602 genes were over-expressed and 1,052 were under expressed among high-risk patients relative to those at low-risk of mortality (**Supplementary Table S4a**). Of note, genes coding for 4 out of the 5 PERSEVERE-II biomarkers - *GZMB*, *CXCL8* (IL-8), *HSPA1B*, and *MMP8* were overexpressed DEGs among high-risk relative to low-risk patients. **Figure 2b** shows the volcano plot highlighting the most differentially expressed genes (DEGs) based on a log2FC threshold of > ± 1 and adjusted p value of <0.001.

**Figure 3:**
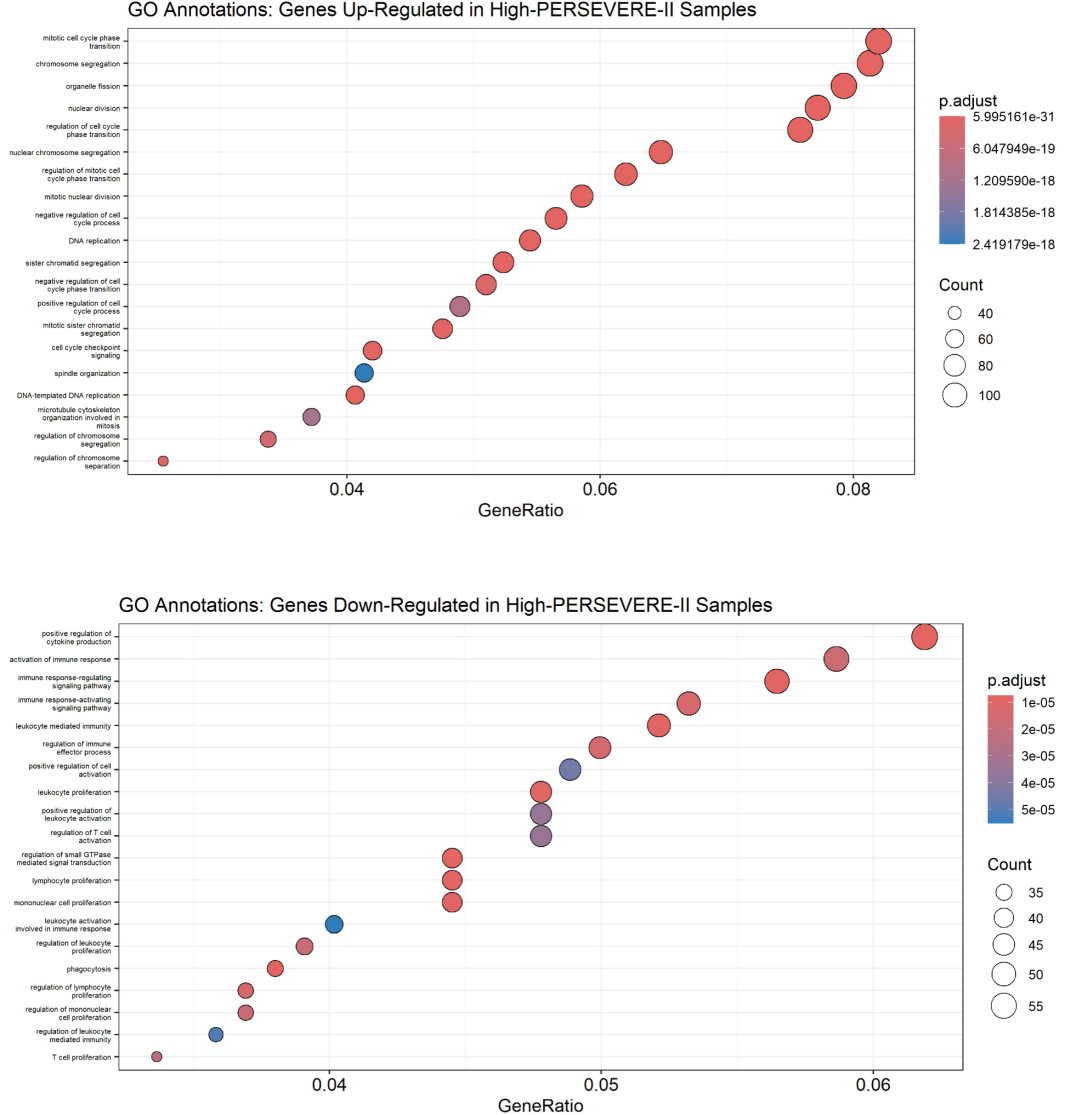
Gene Ontology annotations up-regulated (top panel) and down-regulated (bottom panel) in high-risk children with sepsis suggest the function of differentially expressed genes. A Benjamini-Hochberg adjusted p-value < 0.05 was used as a significance threshold for clusterProfiler analysis.

As shown in **Figure 3**, top panel), genes over-expressed in high-risk samples were involved in the cell cycle, with the most-enriched GO terms being “mitotic cell cycle phase transition,” “chromosome segregation,” “organelle fission,” “nuclear division,” and “regulation of cell cycle phase transition.” The genes repressed among high-risk patients were involved in “positive regulation of cytokine production,” “activation of immune response,” “immune-response regulating signaling pathway,” “immune-response activating signaling pathway,” and “leukocyte-mediated immunity.” Alternatively, GO annotations “T cell proliferation” and “regulation of T cell activation” (**Figure 3**, bottom panel) and KEGG pathways “T cell receptor signaling pathway” and “B cell receptor signaling pathway” (**Supplementary eFigure S3****),** reflective of the adaptive immune response, were repressed among high-risk patients.

### Developing neutrophils contribute disproportionately to host pathobiology in the high-risk strata

**Figure 4:**
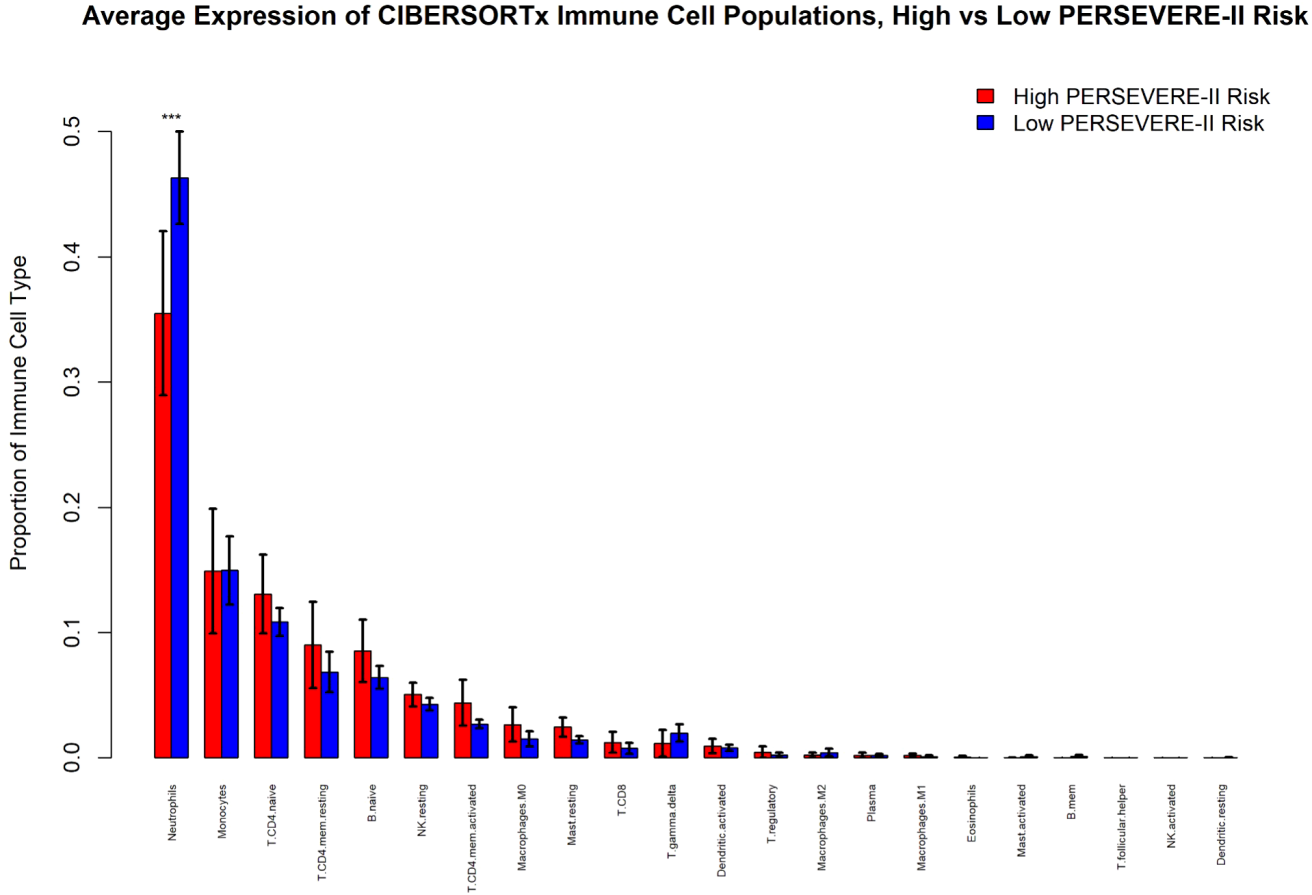
CIBERSORTx analysis of immune cell populations comparing children at high and low risk for sepsis mortality using PERSEVERE-II risk-strata. “***” indicates the 95% confidence intervals between the two conditions are non-overlapping.

**Figure 5:**
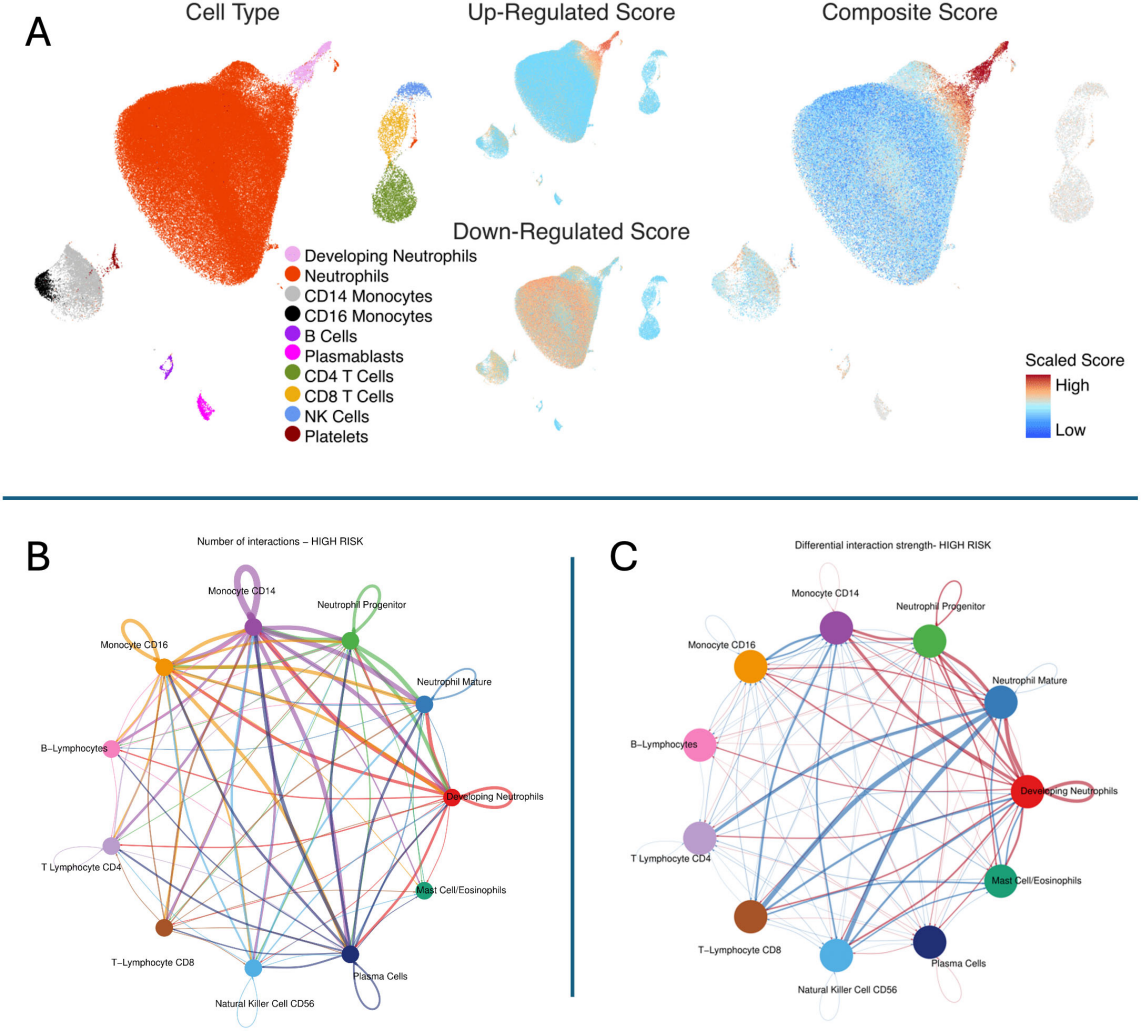
Inference of cell subsets underlying pediatric septic shock mortality risk strata. Top Panel **(A)** The figure shows the Uniform Manifold Approximation and Projection (UMAP) derived from the single-cell transcriptomic dataset from critically ill adults with sepsis published by *Kwok* et al. Cell Type: Ten cell subsets were identified in the single-cell dataset. (1) Developing neutrophils (pink), (2) Mature neutrophils (red), (3) Cluster differentiation (CD) 14 positive monocytes (light gray), (4) CD16 positive monocytes (black), (5) B lymphocytes (deep purple), (6) Plasmablasts (purple), (7) CD4 positive T lymphocytes (moss green), (8) CD8 positive T lymphocytes (yellow), (9) NK, Natural killer cells (blue), and (10) Platelets (brown). Up-Regulated Score: Up-regulated genes among high-risk patients are shown in red. Down-regulated Score: Down-regulated genes among high-risk patients are shown in red. Composite Score: Composite gene score represents geometric mean of upregulated minus downregulated genes among patients belonging to high-risk strata. The gene score was scaled as shown in the legend. Cells in red represent those with a high composite gene score indicating that they contributed predominantly to overexpressed genes among patients with high mortality risk. In contrast, cells in blue represent those with a low composite gene score indicating that they contributed predominantly to genes underexpressed among patients with low mortality risk. Bottom Left **(B)** The plot shows the number of interactions in high-risk patients. Notably, developing neutrophils exhibited a higher number of interactions compared to other cell types, suggesting a key role in the hyperinflammatory response observed in high-risk patients (shown in red). Bottom Right **(C)** The plot represents the differential interaction strength in high-mortality risk patients. Developing neutrophils, monocytes (CD14, CD16), and neutrophil progenitors display stronger interactions in the high-risk group, (shown in red) with developing neutrophils being a central hub of communication, indicative of their critical role in driving the hyperinflammatory response.

We identified that high mortality-risk patients had a lower average fraction of mature neutrophils (35.5%; 95% CI [29.0%, 42.0%]) compared with low mortality-risk patients (46.3%; 95% CI [42.6%, 50.0%]) based on CIBERSORTx analyses shown in **Figure 4**. There was no appreciable difference in the proportion of any of the other 21 cell types imputed by CIBERSORTx. As shown in **Figure 5**, the *Kwok* et al. dataset had 10 cell types from critically ill adult patients with sepsis. Genes upregulated among high-risk patients were expressed primarily by a small population of developing neutrophils. Further, downregulated genes among patients with high mortality risk were expressed primarily by mature neutrophils. We further identified that developing neutrophils exhibited greater number and strength of inter-cellular interactions, specifically among high-mortality risk patients.

### Identification of key transcriptional regulators

Gene lists of WGCNA submitted to ChEA3, identified *LTF*, itself a gene in the Pink Module, as the most enriched TF with a mean rank of 1.0 and 10 overlapping genes including *MMP8, LCN2, and RETN.* Other TFs of interest identified included *KLF1 (Brown Module*, mean rank of 1.6 and 231 overlapping genes*), FOXM1 (Yellow Module*, mean rank of 2.6 and 192 overlapping genes), and *ZNF12* (*Purple Module*, mean rank of 8.0 and 13 overlapping genes). Among the top regulator effect networks associated with high mortality-risk patients, identified through IPA analyses of DEGs, was *CEBPB* with an activation z-score of 5.08 and p-value of 1.11 e^-12^, indicating the degree of overlap in genes from the dataset and those modulated by the particular TF. Other key TFs identified included *TFEB*, *MYC*, and *TBX3.* Consistent with results using the former approach, *FOXM1* was identified to be activated with a z-score of 3.3 *and* p-value of overlap of 5.2 e^-11^ and *KLF1* had an activation z-score of 1.7 and p-value of 1.3 e^-16.^ In contrast, *LTF* and *ZNF12* were not enriched when using causal network analyses in IPA (**Supplementary Table S5**).

## Discussion

In this study we analyzed whole blood transcriptomic profiles from 81 pediatric septic shock patients, including 24 high- and 57 low- mortality-risk patients based on the prospectively validated PERSEVERE-II biomarker stratification tool. Using Weighted Gene Co-Expression Network Analysis (WGCNA), which assigns colors to modules, we identified four gene modules associated with high mortality risk, with the 51-gene “Pink” Module being most strongly correlated. Functional pathways linked to these modules highlighted the roles of innate immune responses and neutrophil degranulation as key factors associated with severe outcomes. Moreover, genes overexpressed in high-risk patients were enriched in neutrophil turnover, while those repressed were related to adaptive immunity. It is also notable that the seven genes identified by WGCNA as being most associated with the high-risk stratum were also identified by DESeq2 as being under-expressed in high-risk patients. These genes (*NHSL2*, *LRMP*, *IL16*, *GLIPR1*, *SRPK2*, *AOAH*, *CALB1*) are among the most under-expressed in high-risk patients (**Supplementary Table S4b**) and warrant further investigation. High-risk patients exhibited a greater contribution of developing neutrophils to gene-expression signatures and fewer mature neutrophils, emphasizing the impact of neutrophil turnover. Lastly, transcription factors identified through complementary approaches resulted in several potential drivers of gene programs for future mechanistic study.

The original PERSEVERE biomarker model was developed by selecting 12 candidate protein biomarkers associated with genome-wide expression profiles differentiating patients based on outcome. Subsequently, CART analyses were used to identify a parsimonious set of 5 protein biomarkers (IL8, HSPA1B, GZMB, MMP8, and CCL3) in addition to patient age to stratify patients (8). The PERSEVERE II model was developed expressly to improve the performance of PERSEVERE among children with septic shock and multiorgan failure (9). Both models have been extensively prospectively validated in cohorts of pediatric septic shock (8–10). Notably, several of the genes (*MMP8, LCN2*, and *RETN*) in the WGCNA module most highly correlated with high mortality risk are either represented in the PERSEVERE risk model or were candidate biomarkers. Moreover, DEG analyses revealed that 4 out of the 5 genes encoding for PERSEVERE-II biomarkers, with the exception of CCL4, were differentially expressed between high- and low-risk patients. The congruence of these data adds confidence in our analyses.

The identification of the contribution of developing neutrophils to patient risk-strata is wholly unsurprising. Recently, *Kwok* et al. used single-cell RNA sequencing to reveal that an adult sepsis gene-expression endotype, Sepsis Response Signature 1 (SRS1), was defined by emergency granulopoiesis (11). Using orthogonal approaches, other groups including our own, have performed latent profile analyses of critically ill adults and pediatric patients with sepsis. Transcriptomic analyses of these patients indicate a key contribution of developing neutrophils to subclass-specific pathobiology (15, 28, 29). Of interest, the highest risk subset of patients is consistently characterized by proliferation of developing neutrophils with concomitant suppression of the adaptive immune system (30–32), resulting in an unchecked hyperinflammatory state. While such a phenomenon has been attributed to the presence of myeloid derived suppressor cells (MDSCs) later in the course of sepsis (33), the mechanistic basis of such crosstalk between the innate and adaptive arms of the human immune system remains to be fully elucidated.

We sought to identify transcription factors (TFs) that simultaneously regulate the expression of numerous genes related to the high-risk mortality strata. Lactotransferrin or Lactoferrin (*LTF*) was predicted to regulate the 51-gene “Pink” module most highly associated with high mortality probability based on WGCNA analyses and ChEA3 TF analyses. Lactoferrin (*LTF*) is an iron-binding glycoprotein that plays a crucial role in immune defense by modulating immune responses, controlling oxidative cell function, and maintaining tissue integrity, thereby limiting pathological damage in response to inflammatory injury and promoting physiological homeostasis (34). Forkhead box M1 (*FOXM1*) and Kruppel-Like Factor 1 (*KLF1*), identified both through WGCNA and DEG-based computational pipelines, are thought to serve as master regulators of DNA damage response (35) and promoting activation of innate immunity through Th1 responses in macrophages (36), respectively. Finally, *CEBPB (*CCAAT Enhancer Binding Protein Beta) identified through DEG and IPA analyses is an established regulator of emergency granulopoiesis (11, 37). While ChEA3 performs better than algorithmic peers in predicting transcription factor association with a set of genes (25) and has been used to identify transcription factors associated with many phenotypes including papillary thyroid cancer (38), infant brain gene expression (39), dermatologic malignancies (40), and mesenchymal stem cells (41), among others (42), these data are correlative. Hypothesis-driven studies focused on these TFs may further shed light on the mechanistic basis of disease and inform development of targeted drugs aimed to ameliorate neutrophil dysregulation.

Our study has several limitations: (1) The sample size of patients with biomarker and transcriptomic data was relatively small, warranting validation in larger datasets to confirm findings and enhance generalizability. (2) The transcriptomic analysis was based on a single blood sample, which limits insights into dynamic gene expression changes that may occur later in the disease course. (3) Gene expression changes may not fully translate to protein levels due to post-translational modifications; integrating high-throughput proteomic data could improve robustness and reveal causal regulatory networks. (4) Single-cell reference data from adults was used to infer cell types, but pediatric-specific data is needed to directly validate findings given age-related differences in sepsis responses. (5) The transcription factor analysis was exploratory, and further studies are needed to confirm the identified regulators’ roles in disease pathology and evaluate their therapeutic potential.

## Conclusions

This study reveals key molecular distinctions in mortality risk for pediatric septic shock patients, as identified by the PERSEVERE-II biomarker risk model. Transcriptomic analyses highlighted innate immune dysregulation, specifically increased neutrophil turnover, and suppressed adaptive immunity among high-risk patients. Developing neutrophils emerged as major contributors to the hyperinflammatory state linked to severe outcomes. Transcription factors such as *LTF*, *FOXM1*, *KLF1*, and *CEBPB* were identified as likely regulators of these gene-expression patterns. These findings provide a foundation for future mechanistic studies and may aid in the development of targeted interventions for high-risk pediatric sepsis patients.

## Data Availability

The data presented in the study are deposited in the NCBI Sequence Read Archive (SRA) repository, accession number PRJNA1358292.
