# Supplementary material for: Neutrophil dysregulation differentiates pediatric septic shock biomarker-based mortality-risk strata: insights from weighted gene co-expression network and transcriptomic analyses
Source: Front Immunol. 2025 Nov 19;16:1663704. doi: 10.3389/fimmu.2025.1663704 (PMC12672347; doi:10.3389/fimmu.2025.1663704)
Supplement: Supplementary file 1 [file DataSheet1.docx]

**Neutrophil Dysregulation Differentiates Pediatric Septic Shock Biomarker Based Mortality Risk Strata: Insights from Weighted Gene Co-expression Network and Transcriptomic Analyses.**

**Supplementary Figures Legend:**

**eFigure 1.** A soft threshold of 12 was chosen for weighted gene co-expression analysis meeting our scale free topology fit goal of R^2 > 0.80 (R^2 = 0.817) and mean connectivity goal of < 100 (mean k = 70.200).

**eFigure 2**: Eigengene expression of modules of interest comparing high- and low-risk patients. Displayed as a boxplot within a violin plot. "***" indicates a Benjamini-Hochberg adjusted p-value < 0.05 for a two-tailed heteroskedastic t-test comparing eigengene expression for high- and low-risk patients.

**eFigure 3:** KEGG annotations up-regulated (top) and down-regulated (bottom) in high-risk children with sepsis suggest the function of differentially expressed genes. A Benjamini-Hochberg adjusted p-value < 0.05 was used as a significance threshold for clusterProfiler analysis. GeneRatio denotes the proportion of genes in the gene list (over- or under-expressed genes) involved in a given functional annotation.


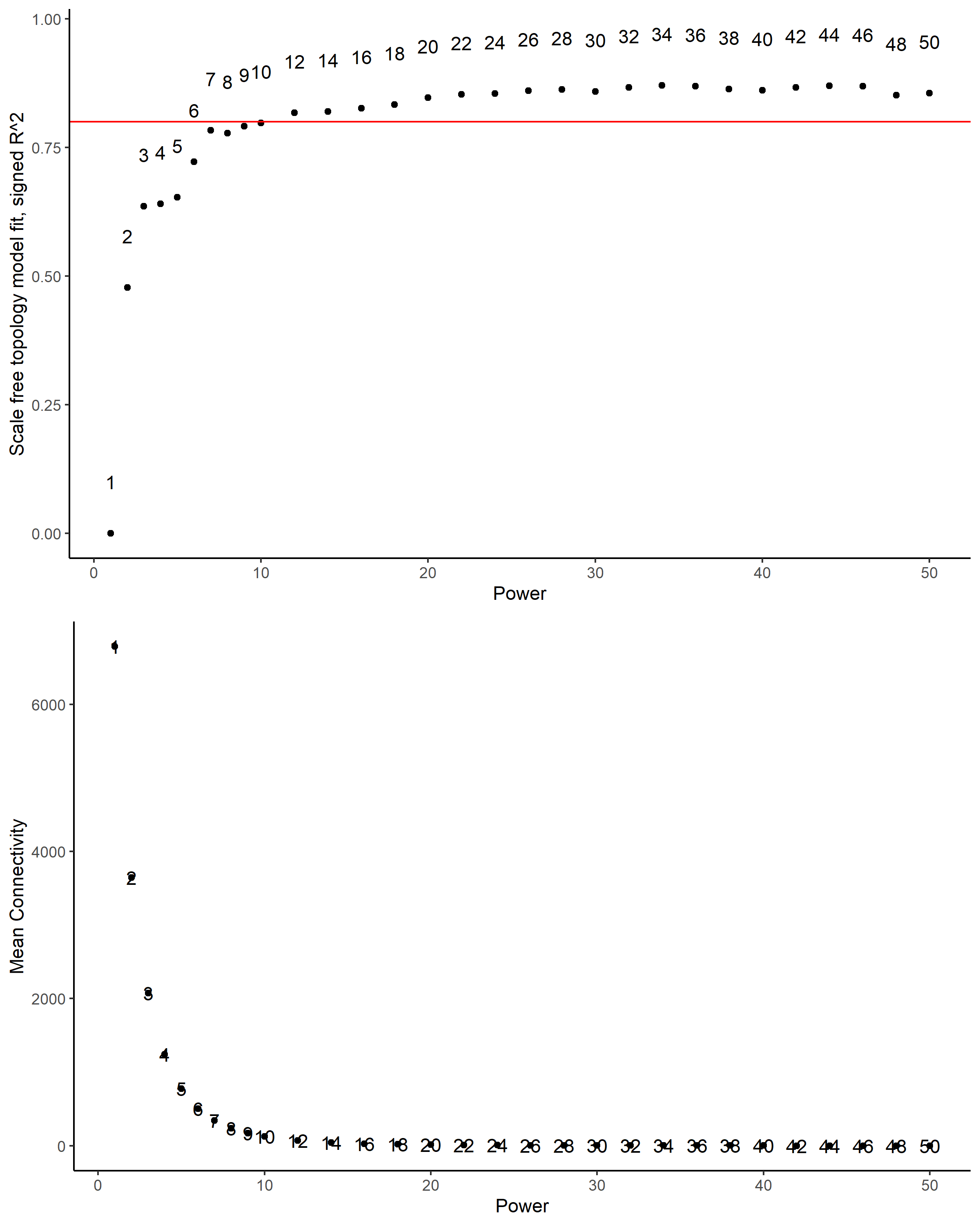
**eFigure 1.**

| \|  \| \| --- \|   **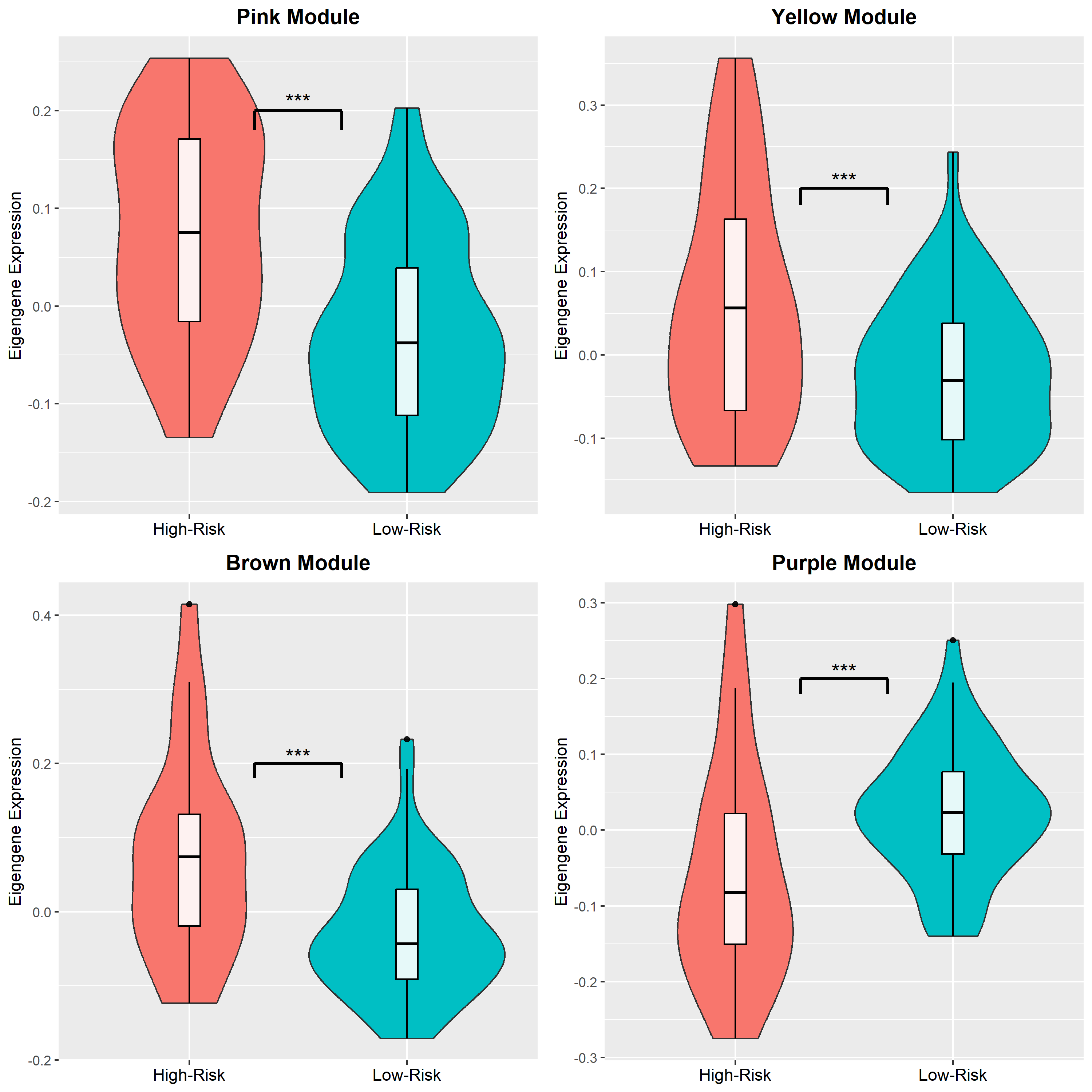eFigure 2.** |  |  |  |  |  |  |  |  |  |  |  |
| --- | --- | --- | --- | --- | --- | --- | --- | --- | --- | --- | --- | --- |
|  |  |  |  |  |  |  |  |  |  |  |  |
|  |  |  |  |  |  |  |  |  |  |  |  |
|  |  |  |  |  |  |  |  |  |  |  |  |
|  |  |  |  |  |  |  |  |  |  |  |  |
|  |  |  |  |  |  |  |  |  |  |  |  |
|  |  |  |  |  |  |  |  |  |  |  |  |
|  |  |  |  |  |  |  |  |  |  |  |  |
|  |  |  |  |  |  |  |  |  |  |  |  |
| **eFigure3.** |  |  |  |  |  |  |  |  |  |  |  |
| 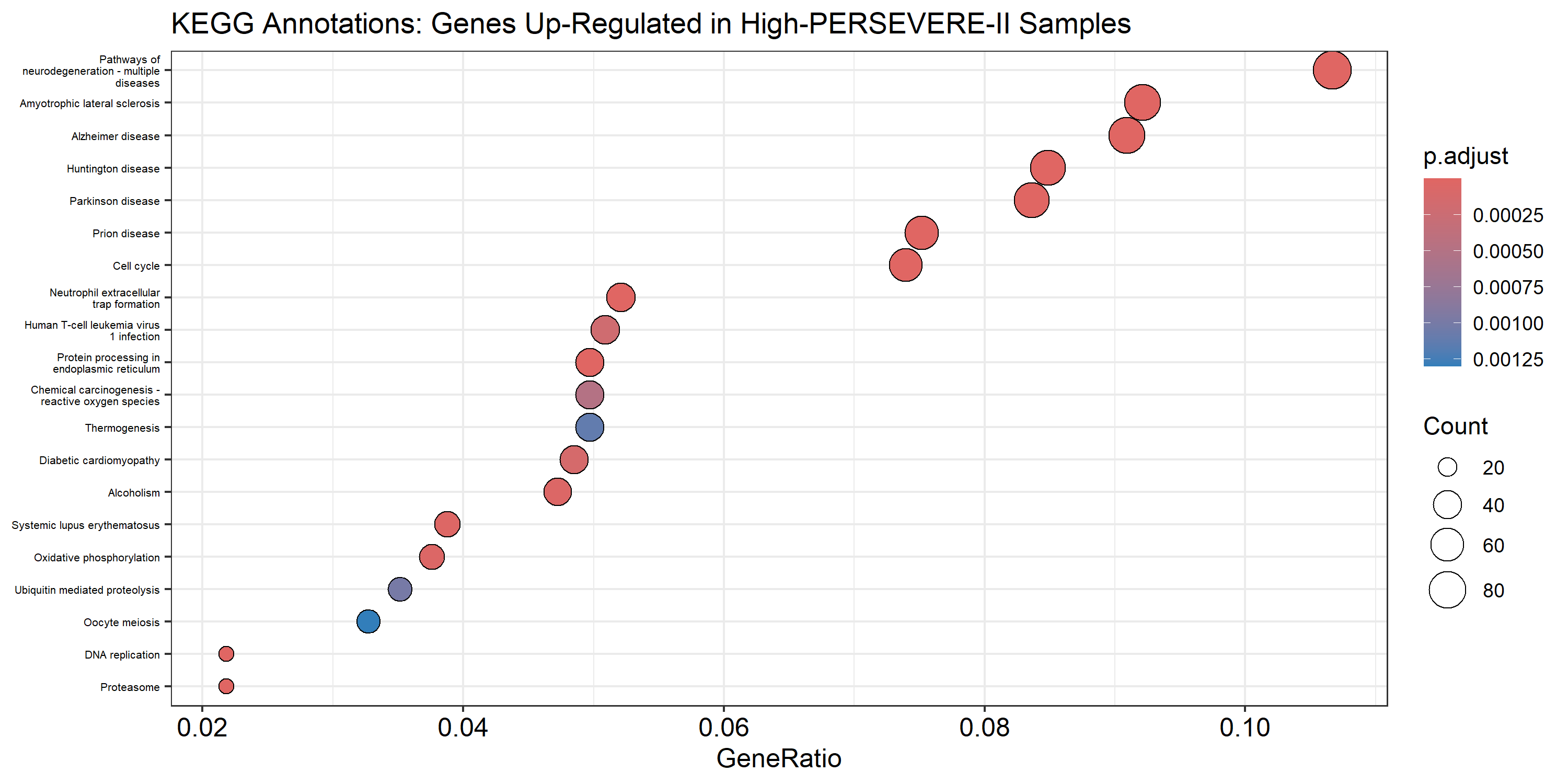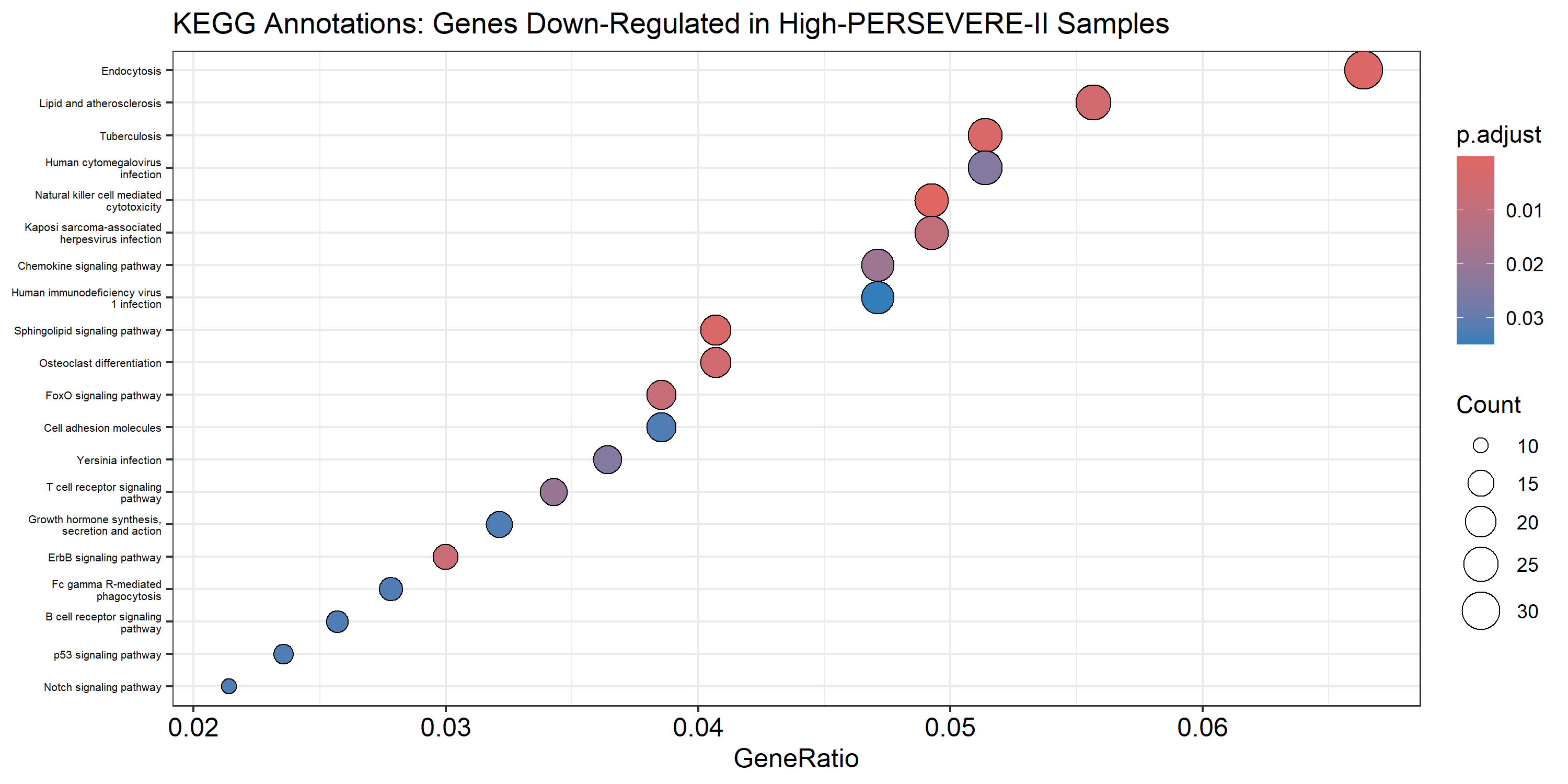 |  |  |  |  |  |  |  |  |  |  |  |
